# Targeted Therapies in Surgical Treatment of Lymphedema: A Systematic Review

**DOI:** 10.7759/cureus.5397

**Published:** 2019-08-16

**Authors:** Antonio J Forte, Daniel Boczar, Maria T Huayllani, Gabriela J Cinotto, Sarah McLaughlin

**Affiliations:** 1 Plastic Surgery, Robert D. and Patricia E. Kern Center for the Science of Health Care Delivery, Mayo Clinic Florida, Jacksonville, USA; 2 Surgery, Robert D. and Patricia E. Kern Center for the Science of Health Care Delivery, Mayo Clinic Florida, Jacksonville, USA

**Keywords:** lymphedema, targeted therapy, chronic lymphedema, lymph nodes

## Abstract

Although physiologic surgeries for lymphedema (i.e., lymphovenous bypass, vascularized lymph node transplantation) are becoming well established, unpredictable outcomes have still been reported in some studies. Therefore, authors have investigated ways to improve these surgery outcomes. The goal of our study was to conduct a comprehensive systematic review of targeted therapy administration in the surgical treatment of lymphedema. We conducted a comprehensive systematic review of the published literature on targeted therapies associated with lymphedema surgery using the PubMed database. Eligibility criteria excluded papers that reported surgical treatment of lymphedema without the use of targeted therapies and also papers describing targeted therapies in nonsurgical treatment of lymphedema. Abstracts, presentations, reviews, and meta-analyses were also excluded.

Extracted data included the year of study, country, lymphedema model, surgical technique, targeted therapy agent, therapy delivery, findings, and outcomes. From 823 potential papers found in the literature, 10 studies fulfilled the eligibility criteria. All papers were experimental, and most of them on small animal model (7/10). Different targeted therapies were proposed, but all of them were associated with lymph node transplantation. The most common targeted therapy proposed mechanism was growth factor delivery (8/10). However, one paper used adipose-stem cell, and one paper proposed the use of sterile inflammation. The pooled publications assessing targeted therapy administration in the surgical treatment of lymphedema demonstrate encouraging data for positive outcomes. To date, all studies were experimental and related to lymph node transfer.

## Introduction and background

Lymphedema is a chronic lymphatic condition frequently related to cancer treatment (i.e., radiation, lymphadenectomy) in developed countries. It is estimated that 5 to 6 million people have lymphedema in the United States, and the incidences described in the literature are high, such as one in every six patients undergoing solid tumor treatment [1]. However, in spite of mainstream thinking, most patients develop lymphedema only months after the lymphatic injury, demonstrating that the physiopathology behind it involves further inflammatory steps, such as fibrosis (i.e., Th2-inflammatory response), where the parenchyma is replaced by scar tissue [2-4].

Targeted treatments are of interest in lymphedema treatment literature. Anti-inflammatory treatments targeting Th2-inflammatory responses have been described [5-7]. Moreover, experimental studies of secondary lymphedema have demonstrated that vascular endothelial growth factor (VEGF) C is an effective promoter of lymphangiogenesis, with potential to reduce tissue edema [8-9]. Studies also have shown the potential of stem cells to differentiate into lymphatic endothelial cells [10]. However, clinical translation of such therapies has been an object of concern, considering the potential risk of metastasis in cancer patients [9].

Lymphedema surgery advanced considerably through the advent of physiologic methods, which aim to restore lymphatic function and continuity. These methods use two microsurgical techniques: 1) lymphovenous bypass (LVB), which connects a lymphatic vessel with a regional vein and is a safe procedure that is effective in early-stage lymphedema [11-14], and 2) vascularized lymph node transplantation (VLNT), which transfers a healthy lymph node to the lymphedematous limb, only reconnecting its blood supply and leaving the afferent and efferent lymphatic vessels to regenerate via spontaneous lymphangiogenesis [15-16].

Interestingly, while physiologic surgeries for lymphedema are becoming well established, some studies are still reporting unpredictable outcomes [17]. Therefore, investigating ways to improve lymphedema surgery outcomes are extremely relevant, and efforts have been made to study the association of LVB and VLNT techniques [17]. Furthermore, some authors have taken advantage of the well-described immunologic physiopathology of lymphedema to postulate that targeted therapies may be used to improve surgery outcomes. Thus, we conducted a systematic review of publications assessing targeted therapy administration in the surgical treatment of lymphedema.

## Review

Search strategy

**Figure 1:**
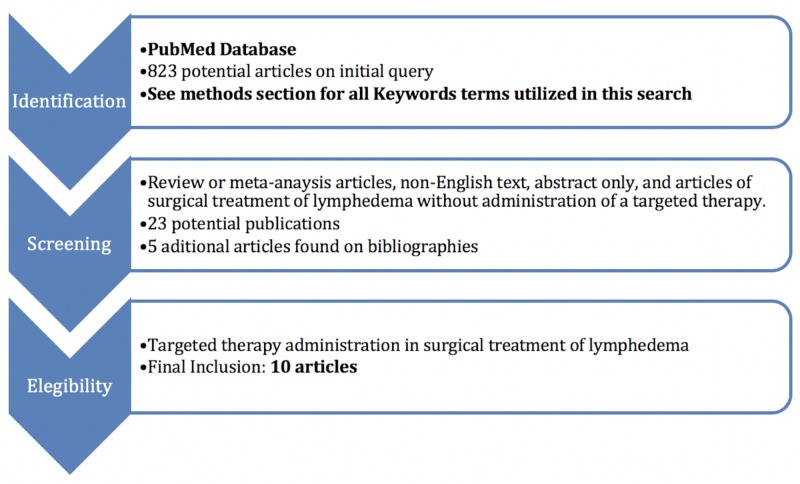
Preferred Reporting Items for Systematic Reviews and Meta-Analyses (PRISMA) diagram

Two reviewers (D.B., M.T.) conducted independently searches using the PubMed database without timeframe limitations, initially through title and abstract screen and then by full-text review. Disagreements regarding article identification and final selection for the inclusion of the literature were resolved by another reviewer (A.J.F). The search was done using the following keywords: ((((Lymphedema) AND Lymphovenous anastomosis)) OR ((Lymph node transfer) AND Lymphedema)) OR ((Lymphedema) AND Growth Factor). The bibliographies of the studies that fulfilled the study eligibility criteria were also examined, looking for articles not present in our initial search. This study followed the guidelines outlined in the Preferred Reporting Items for Systematic reviews and Meta-Analyses (PRISMA) flowchart in Figure 1.

Selection criteria

Eligibility criteria included studies reporting data from the use of targeted therapies in the surgical treatment of lymphedema. Therefore, we excluded papers that reported surgical treatment of lymphedema without the use of targeted therapies and also papers describing targeted therapies in non-surgical treatment of lymphedema. Abstracts, presentations, reviews, and meta-analyses were also excluded. 

Data extraction and processing

Extracted data included the year of study, country, lymphedema model, surgical technique, targeted therapy agent, therapy delivery, findings, and outcomes. Data extraction from articles, tables, and figures was performed by two reviewers (D.B., M.T.), with the accuracy of data entry confirmed by an additional reviewer (A.J.F). 

Study characteristics

From 823 potential papers found in the literature, 10 studies fulfilled the study eligibility criteria as seen in Figure 1 and Table 1. Targeted therapies for surgical treatment of lymphedema were described by groups from different countries, but half of them (5/10) were from Finland. All papers were experimental; most on rats (small animal model, 7/10), and the others on pigs (large animal model, 3/10). Different types of targeted therapies in the surgical treatment of lymphedema were proposed, but all of them were associated with lymph node transplantation. No papers were identified in the literature about targeted therapies applied to LVB. Eight papers reported targeted therapies with VLNT and two with transplantation of avascular autologous lymph node fragments. The mechanism of targeted therapy most commonly proposed was growth factor delivery (7/10). Other targeted therapies proposed were adipose-stem cells, platelet-rich plasma, and sterile inflammation. 

**Table 1:** Summary of the Study Findings VEGF, Vascular Endothelial Growth Factor; VLNT, Vascularized Lymph Node Transfer; PRP, Platelet-Rich Plasma.

Author	Year	Country	Type of study	Model	Technique	Mechanism	Agent	Delivery	Findings	Conclusion
Tervala et al.	2015	Finland	Experimental	Rat	VLNT	Growth factor	VEGF-C, VEGF-D, VEGF-C156S, VEGF-A	Local Adenoviral vectors	Lymphangiogenesis (VEGF-C and VEGF-D); Improved lymphatic function (VEGF-C); Better lymph node survival compared to control and VEGF-A (VEGF-C, VEGF-D, VEGF-C156S). VEGF-C provided greatest therapeutic results compared to other VEGFs	VEGF-C is the preferred growth factor therapy of lymphedema
Hayashida et al.	2017	Japan	Experimental	Rat	VLNT	Autologous tissue	Adipose-Derived Stem Cells	Local injection	Mice that received combined treatment (VNLT + Adipose-derived stem cell) had better percentage of improvement and percentage deterioration, increased lymphatic vessels with LYVE-1 immunoreactivity. Moreover, they developed lymph node metastases more quickly than the control group when injecting B16 melanoma cells.	Combine VNLT and adipose-derived stem cell may be a effective treatment for secondary lymphedema
Lahteenvuo et al.	2011	Finland	Experimental	Pig	VLNT	Growth factor	VEGF-C, VEGF-D	Local Adenoviral vectors	Post-operative lymphatic drainage was superior in VEGF-C and VEGF-D treated pigs. VEGF-C and VEGF-D induced growth of functional lymphatic vasculature. Pigs that received VEGF-C had better preservation of the transferred lymph node structure. VEGF-D transiently increased seroma by increasing vascular permeability	VLNT associated with gene therapy can repair lymphatic circulation in large animals, which supports basis for future clinical trials. Brief VEGF-C gene expression through adenovirus can promote formation of stable collecting lymphatic vessels
Sommer et al.	2011	Germany	Experimental	Rat	VLNT	Growth factor	VEGF-C	Local injection 3 times after transplantation (1st, 2nd and 5th post-operative day)	Histological pattern of regenerated lymph nodes: 74% VEGF-C group (14/19) vs. 59% control group (13/22); Connection of VNLT with superficial lymphatic vessels of the leg: 36% VEGF-C group (5/14) vs. 15% control group (2/13)	Injection of VEGF-C in the VLNT area promotes improved outcomes on lymphatic reconnection and histological regeneration
Tammela et al.	2007	Finland	Experimental	Rat	VLNT	Growth factor	VEGF-C, VEGF-D	Local Adenoviral vectors	Both VEGF-C and VEGF-D induced robust growth of the lymphatic capillaries. Incorporation to pre-existing lymphatic network: 82% in VEGF-C-treated mice (9/11) vs. 22% of control group (2/9). VEGF-C-treated lymph nodes developed both afferent and efferent connections with the host lymphatic circulation. More over, they were bigger than control group (control group lymph nodes that were not in contact with the host lymphatic system regressed). Injection of human lung carcinoma cells subcutaneously demonstrated that these cells were trapped in 80% of VEGF-C-treated lymph nodes (8/10) vs. 17% of control group (1/6)	VLNT associated with growth factor therapy had improved outcomes and functional immunological barrier against tumor metastases
Schindewolffs et al.	2014	Germany	Experimental	Rat	Avascular Autologous Lymph Node Fragments	Growth factor	VEGF-C	Local injections	There was a correlation between high doses of VEGF-C and lymphatic regeneration. Application in early postoperative and at the medial thigh seems to promote better results, although not statistically significant	Lymph node fragments transplant associated with VEGF-C might be useful in treatment of secondary lymphedema
Honkonen et al.	2013	Finland	Experimental	Pig	VLNT	Growth factor	VEGF-C	Local Adenoviral vectors (intranodally vs. perinodally)	Compared to control (saline), both intranodally and perinodally injection induced lymphangiogenesis and helped to preserve transplanted lymph node structure. Intranodal injection had as adverse effect the accumulation of Macrophages inside the node	Perinodal delivery of adenoviral VEGF-C is the better route of delivery for future clinical studies
Joseph et al.	2014	USA	Experimental	Rat	Avascular Autologous Lymph Node Transplant	Sterile inflammation	Immune adjuvant (Complete Freund's Adjuvant, CFA; 2% ovalbumin, OVA)	Local injection	Compared to control (no-sterile-inflammation) or sterile-inflammation before lymph node transplant groups, the group of sterile inflammation delivered after transplantation had a >2-fold increase in lymphatic function, a increased lymphangiogenesis, and a more functional lymphatics. Moreover, inside the nodes, this group also had a expansion of B-cell zones and decreased percentage of T-cells	Sterile inflammation after lymph node transplantation promotes preservation of lymph node structure.
Visuri et al.	2015	Finland	Experimental	Pig	VLNT	Growth factor	VEGF-C, VEGF-C156S	Local Adenoviral vectors	Both VEGF-C and VEGF-C156S induced lymphangiogenesis. However, lymphangiogenesis and lymph node preservation was superior with VEGF-C. Enlargement of blood vessels with VEGF-C was not correlated to increased wound exudate through vascular permeability.	VEGF-C is the preferred growth factor therapy of lymphedema
Hadamitzky et al.	2008	Germany	Experimental	Rat	Avascular Lymph nodes transplant	Autologous tissue	PRP	Local Injection	Strongly contrasted small secondary follicles in the Para cortical region of the transplanted lymph nodes (sign of proliferative reaction) were seen after delivery of PRP, compared to the control group.	PRP could improve regeneration of new lymphatic vessels in transplanted lymph nodes.

VEGF-C 

Delivery of VEGF-C was assessed in seven papers, all agreeing that its administration promotes positive results on lymphedema compared to controls. The most common method of VEGF-C delivery was a local injection of adenoviral vectors (6/7). Lahteenvuo et al. demonstrated in pigs that a brief VEGF-C gene expression through adenovirus was enough to promote the formation of stable collecting lymphatic vessels [18]. In their study, adenoviral VEGF-C transfer promoted a 50% increase in lymphatic flow compared to control [18]. Sommer et al. studied in rats the effect of local injection of VEGF-C VLNT postoperative days 1, 2, and 5 [19]. They noticed a histologic pattern of regenerated lymph nodes in 74% of the VEGF-C group (14/19) versus 59% of the control (13/22), and connection of VNLT with superficial lymphatic vessels of the leg in 36% of the VEGF-C group (5/14) versus 15% of the control (2/13) [19].

Other growth factors

Some authors compared VEGF-C with other growth factors (e.g., VEGF-A, VEGF-C156S, and VEGF-D), demonstrating that VEGF-C provided the greatest therapeutic results and justifying its further study in combination with surgical treatment of lymphedema. Authors who studied the effects of VEGF-D associated with VLNT demonstrated that it could induce lymphangiogenesis [18,20-21]. However, one study pointed out that VEGF-D transiently increased seroma by increasing vascular permeability and had inferior preservation of the transferred lymph node structure compared to the group treated with VEGF-C [18]. Interestingly, one study proposed a local injection of platelet-rich plasma, which is a potential autologous source of growth factors (e.g., platelet-derived growth factor, transforming growth factor β, platelet-derived angiogenesis factor) [22]. Their results demonstrated that platelet-rich plasma can increase the survival rate of lymph node fragments transplanted in rats.

Sterile inflammation

Taking into consideration the potential risk of metastasis by using growth factor therapy in lymphedema treatment, Joseph et al. proposed local sterile inflammation inducers, which are used in vaccines to boost its immunization potential [17]. The authors demonstrated that, compared to control (no-sterile-inflammation) and sterile inflammation before VLNT, the group of sterile inflammation delivered after transplantation had more than 2-fold increase in lymphatic function, an increased lymphangiogenesis, and a more functional lymphatic vasculature. Moreover, inside the nodes, this group had an expansion of B-cell zones and decreased percentage of T cells [17].

Metastasis assessment

Experiments using tumor cells with high potential of lymphatic metastasis are useful to assess lymphatic function. Two authors have assessed metastasis in targeted therapy in lymphedema treatment. Tammela et al. studying VEGF-C and VEGF-D delivery by adenovirus in rats that underwent VLNT, demonstrated through subcutaneous injection of human lung carcinoma cells, that cancer cells were trapped in 80% of VEGF-C-treated lymph nodes (8/10) versus only 17% in the control group (1/6) [21]. They concluded that VLNT associated with growth factor therapy had improved outcomes and functional immunologic barrier against tumor metastases [21]. Hayashida et al. assessing the local injection of adipose-derived stem cells in rats that underwent VLNT, noticed that lymph node metastases happened more quickly in the group treated with stem cells compared to control when injecting B16 melanoma cells [23]. At twenty-one days following melanoma cell injection, all the rats treated with stem cells presented lymph node metastasis (3/3) compared to only 33% of the control group (1/3). Moreover, this group also presented metastatic skin tumors on the trunks, which were not found in the control group. At 25 days following melanoma injection, all the rats that received stem cells died due to tumor progression. The authors suggested that adipose-derived stem-cell therapy has the potential to promote recanalization and re-anastomoses of lymphatic vessels on VLNT, improving its capacity to drain the lymphatic fluid [23].

Discussion

In this systematic literature review, we have shown that different mechanisms of targeted therapies in association with lymphedema surgery have been proposed with promising experimental results. The literature of lymphedema pathogenesis and treatment has been increasing considerably over the years. VLNT and LVB are considered functional lymphedema surgeries, since they attempt to improve the local lymphatic circulation. While surgeries to treat lymphedema are generally accepted now, some studies still have reported unpredictable outcomes for them, varying from excellent outcomes to no benefit [17]. Moreover, these procedures are still indicated mainly for patients with early-stage lymphedema. Efforts to improve consistency and outcomes of these functional surgeries are being made in two main directions. In one direction, authors have started to describe the combination of lymph node transplantation and LVB, which has been demonstrating promising results [24]. In the other direction, authors have taken advantage of the breadth of knowledge regarding lymphedema pathogenesis to propose a combination of surgery with targeted therapies, modulating immune response and lymphangiogenesis. To our knowledge, this study is the first systematic literature review assessing the use of targeted therapies (not limited to growth factors) associated with lymphedema surgery.

From this literature review, we noticed that all efforts to propose targeted therapies in lymphedema surgery were focused on lymph node transplantation. The reason behind this is probably the fact that this procedure is dependent on lymphangiogenesis since it employs microsurgical anastomosis only to reconnect the blood supply. Therefore, its result is dependent on the preservation of the transplanted lymph node architecture and the lymph node’s capacity to reconnect with the local lymphatic circulation through the production of endogenous lymphangiogenesis cytokines [15-17].

Although the first targeted therapy associated with lymphedema surgery was proposed in 2007 by Tammela et al., to date, no clinical studies have been published [21]. Most of the studies were done in small animals (rats), and only a few were done in large animals (pigs). What is probably behind the slow advancement into clinical studies is the association of lymphangiogenic growth factors with tumor growth and metastasis [25-26], since lymphedema in developing countries is mainly secondary to oncologic surgical procedures, there is a high concern for using growth factors in these patients. Interestingly, two papers included in our review went further, assessing the potential for metastasis by injecting melanoma and lung cell carcinoma tumors cells into their animal subjects [21,23]. After injection of B16 melanoma cells, the authors who proposed adipose-stem cell therapy noticed the presence of lymph node and skin metastases earlier in the treated group compared to the control [23]. Interestingly, the study of injected human lung carcinoma cells in VEGF-C-treated subjects demonstrated that tumors cells were trapped more often in their treated group, and suggested that this was indicative of an improved functional immunologic barrier by the transplanted lymph nodes against tumor metastasis [21].

Studies have suggested targeted therapies in lymphedema treatment beyond growth factors. For example, it is well accepted that modulation of Th2 cytokines is useful in lymphedema treatment by promoting anti-inflammatory and antifibrotic action without increasing the risk of tumor metastasis [24]. Avoiding the use of growth factors, Joseph et al. proposed the induction of sterile inflammation as a targeted therapy in lymphedema surgery [17]. They employed the complete Freund adjuvant and ovalbumin, substances used to increase a vaccine’s immunization potential [27]. They noticed in the treated group an increased number of B cells (inducers of lymphangiogenesis) and decreased number of T cells (inhibitors of lymphangiogenesis), causing an improvement of lymphangiogenesis and recovery of lymphatic function [17].

We do recognize the presence of several limitations to our study, common to systematic reviews. Studies included in this review were published in the English language. Furthermore, there is a potential for bias in interpreting the data reported in each study. We recognize that Lu et al. performed an experimental study in which adenoviral VEGF-C was delivered to a transferred lymph node [28]; however, the full version of the paper was not found in English, therefore, not fulfilling the inclusion criteria of this study. Despite these limitations, we feel that our study reports valuable pooled data, particularly pertaining to mechanisms of targeted therapies already proposed in lymphedema surgery, which can guide future studies to advance the field. Currently literature is focused on VEGF-C [18-21,29-31].

## Conclusions

The pooled publications assessing targeted therapy administration in the surgical treatment of lymphedema demonstrate encouraging data for positive outcomes. To date, all studies have been experimental and related to lymph node transplantation. Most of the studies proposed growth factor therapies, but lymphangiogenesis without delivering cytokines was also possible through sterile inflammation and adipose-derived stem cells.
